# Genome-Wide Characterization of VDAC Gene Family in Soybean (*Glycine max* L.) and In Silico Expression Profiling in Response to Drought and Salt Stress

**DOI:** 10.3390/plants14142101

**Published:** 2025-07-08

**Authors:** Muhammad Muneeb Ullah, Muqadas Aleem, Muhammad Mudassar Iqbal, Awais Riaz, Ainong Shi

**Affiliations:** 1 Department of Horticulture, University of Arkansas, Fayetteville, AR 72701, USA; ashi@uark.edu; 2 Department of Plant breeding and Genetics, University of Agriculture, Faisalabad 38000, Pakistan; mudassariq5@gmail.com; 3 Department of Crop, Soil and Environmental Sciences, University of Arkansas, Fayetteville, AR 72701, USA; ariaz@uark.edu

**Keywords:** soybean, genome-wide, VDAC, drought, salt stress

## Abstract

Soybean (*Glycine max* L.) is grown worldwide to obtain edible oil, livestock feed, and biodiesel. However, drought and salt stress are becoming serious challenges to global soybean cultivation as they retard the growth of soybean plants and cause significant yield losses. Voltage-dependent anion-selective channel (VDAC) proteins are well-known for their role in drought and salt tolerance in crop plants. In this study, we identified 111 putative VDAC genes randomly distributed in genomes of 14 plant species, including cultivated soybean (*Glycine max*) and wild soybean (*Glycine soja*). The comparative phylogenetic studies classified these genes into six different clades and found the highest structural similarities among VDAC genes of *G. max* and *G. soja*. From the conserved domain database, porin-3 (PF01459) was found to be the conserved domain in all VDAC proteins. Furthermore, gene annotation studies revealed the role of *GmaVDAC* proteins in voltage-gated anion channel activity. These proteins were also found to interact with other proteins, especially mitochondrial receptors. A total of 103 miRNAs were predicted to target fifteen *GmaVDAC* genes. In *G. max*, these genes were found to be segmentally duplicated and randomly distributed on twelve chromosomes. Transcriptomic analysis revealed that the *GmaVDAC18.2* gene showed overexpression in root nodules, whereas the *GmaVDAC9.*1, *GmaVDAC18.1*, and *GmaVDAC18.2* genes showed overexpression under drought and salt stress conditions.

## 1. Introduction

Soybean (*Glycine max* L.) is a widely grown oilseed crop which accounts for 58% of global edible oil production and satisfies approximately 69% of the demand for protein meal [1]. Moreover, the rhizobia inside the root nodules of soybean plants can fix atmospheric nitrogen and thus improve the nitrogen content of the soil. However, the drought and salt stress due to climate change adversely affect the growth and development of soybean [2]. These conditions negatively impact various physiological processes and create an ionic imbalance in the cell, thus exacerbating the production of reactive oxygen species [3], which in turn pose oxidative stress. Ultimately, this leads to low soybean yield, poor oil quality, and decreased nodulation. Thus, the cell tends to maintain its internal homeostatic state by regulating ionic balance and scavenging excessive ROS [4].

Voltage-dependent anion-selective channel (VDAC) genes have been reported to regulate drought and salt stress in plants. VDAC proteins are major mitochondrial proteins (also called mitochondrial porins) that are found in all eukaryotic organisms, namely plants, animals, and fungi [5]. These cover about thirty percent of the surface area of the outer membrane of mitochondria and give a sieve-like structure to it [6]. These are highly conserved transmembrane β-barrel proteins involved in maintaining cellular homeostasis by regulating metabolite transport, especially chloride ions, between the cytoplasm and mitochondria in drought and salt stress conditions. In addition, these proteins also regulate the communication between the cytoplasm and mitochondria by interacting with other proteins [3].

VDAC proteins are also involved in regulating the programmed cell death (PCD) that results from mitochondrial-mediated apoptosis [7]. Under conditions of abiotic stress such drought and salinity, plant cells generally experience an accumulation of reactive oxygen species (ROS) which can trigger PCD and ultimately cause cellular damage [8]. To cope with such conditions, plant cells activate ROS-detoxifying enzymes which scavenge the excessive ROS and help plants survive the stress [9]. However, oxidative stress can damage the ROS-scavenging enzymes and further complicate the survival process. Interestingly, it has been observed that a member of the thioredoxin family, i.e., nucleoredoxin 1 in Arabidopsis (*AtNRX1*), protects the catalase (H_2_O_2_-scavenging enzymes) from ROS-induced oxidative stress [10,11]. A previous study has reported an interaction between *AtVDAC3* and *AtTrxm2* that regulates the level of H_2_O_2_ in plant cells. Thus, the function of VDAC proteins in response to abiotic stress is highly related to thioredoxin family proteins. Another study identified an interaction between *TaVDAC1-B* and Nucleoredoxin-D1 (*TaNRX-D1*). Under salinity, *TaVDAC1-B* overexpression led to an increased *AtNRX1* expression and superoxide dismutase activity, reducing superoxide radical accumulation. Conversely, under drought stress, *TaVDAC1-B* overexpression decreased *AtNRX1* expression and superoxide dismutase activity, increasing superoxide radical accumulation [12].

Some studies have reported the evolutionary history, structural and functional characterization, and role in abiotic stress tolerance of the VDAC gene family in different crops such as *Arabidopsis* [13], beans [14], *Medicago* and tobacco [15], pearl millet [16], rice [17], wheat [4], etc. However, to date, the role of the VDAC gene family under drought and salt stress has remained unknown in soybean. Therefore, this study investigates the structural characterization and evolutionary relationships of VDAC genes in *Glycine max* L. along with their functional characterization in response to drought and salt stress based on the RNA-seq data available from previous studies.

## 2. Results

### 2.1. Identification, Distribution, and Renaming of VDAC Genes

We identified 111 non-redundant VDAC genes that are randomly distributed in the genomes of 14 plant species. Fifteen VDAC genes were found in both *G. max* and *G. soja*, whereas five VDAC genes were found in *A. thaliana* (Supplementary Table S1). For convenience, the gene IDs were renamed in such a way that the first three letters indicate the abbreviated botanical name of each crop, followed by the abbreviated name of the gene family. Of the last two numbers, the one before the placeholder represented the nth chromosome, whereas the other represented the nth VDAC gene on that chromosome [2]. For example, in our case, *GmaVDAC13.2* showed that the 2nd VDAC gene lies on the 13th chromosome in the *G. max* genome. We did not consider any sequence of less than 150 bp, encoding a truncated protein, and a scaffold [18].

### 2.2. Conserved Domain, Localization, and Physico-Chemical Properties of VDAC Proteins

We found PF01459 as the Hidden Markov Model (HMM) indicating a conserved domain (Porin-3) of VDAC proteins. All the peptide sequences devoid of this domain and containing an incomplete domain were not selected for phylogenetic analysis. The sub-cellular localization analysis revealed the presence of all VDAC proteins in the mitochondria of the cell.

The physico-chemical properties of *GmaVDAC* and *GsoVDAC* include peptide length, molecular weight, isoelectric point, and GRAVY (Grand average of hydropathy) score (Supplementary Table S1). The number of amino acids varied from 245 (*GmaVDAC5.1* and *GmaVDAC17.2*) to 333 (*GmaVDAC14.1*) in *GmaVDAC* proteins, whereas this value varied from 245 (*GsoVDAC5.1*) to 277 (*GsoVDAC13.1*, *GsoVDAC14.1*, and *GsoVDAC17.1*) in *GsoVDAC* proteins. The molecular weights (kDa) ranged from 26,545 (*GmaVDAC5.1*) to 35,938 (*GmaVDAC14.1*) in *GmaVDAC* proteins, whereas in *GsoVDAC* proteins, it ranged from 26,545 (*GsoVDAC5.1*) to 29,961 (*GsoVDAC17.1*). Similarly, the isoelectric point varied from 5.96 (*GmaVDAC5.1*) to 9.43 (*GmaVDAC14.1*), and the GRAVY score varied from *−*0.241 (*GmaVDAC13.1*) to 0.024 (*GmaVDAC17.2*) in *GmaVDAC* proteins. However, in *GsoVDAC* proteins, the values were found to range from 5.96 (*GsoVDAC5.1*) to 9.29 (*GsoVDAC11.1*) for the isoelectric point, and −0.24 (*GsoVDAC13.1*) to 0.024 (*GsoVDAC17.2*) for the GRAVY score. One-hundred-percent similarity was found between *GmaVDAC5.1* and *GsoVDAC5.1* proteins for peptide length, molecular weight, pI, and GRAVY score.

### 2.3. Classification and Phylogenetic Analysis

**Figure 1:**
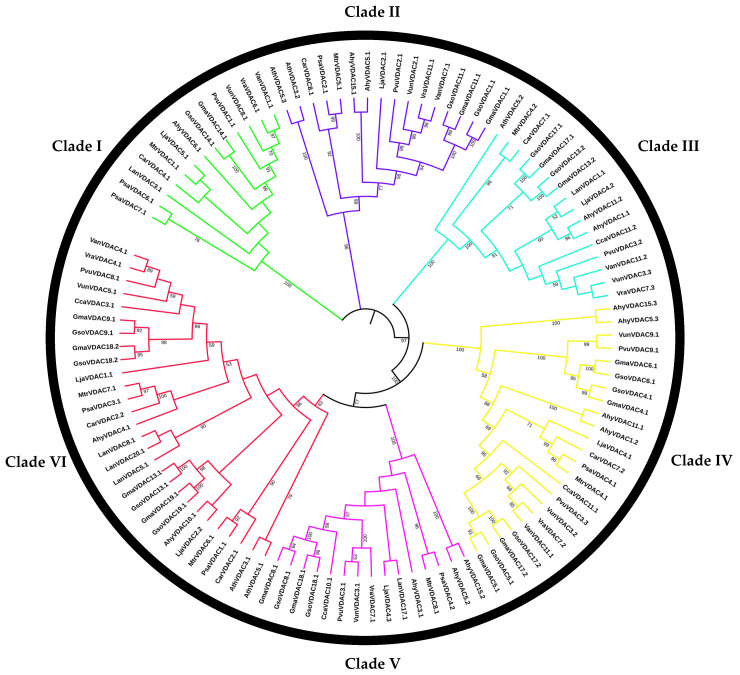
Evolutionary relationship of VDAC genes. A comparative phylogenetic tree of 111 VDAC genes, divided into six clades (I to VI), identified in 14 plant species. Clade I = 13 genes; clade II and III = 16 genes each; clade IV = 23 genes; clade V = 15 genes; clade VI = 28 genes.

The topology of the comparative phylogenetic tree of 111 VDAC genes indicated the distribution of VDAC genes of the concerned species into six different clades (Figure 1). We found 13 genes and one pair of orthologs (*GmaVDAC14.1*/*GsoVDAC14.1*) in clade I, 16 genes and two pairs of orthologs (*GmaVDAC11.1*/*GsoVDAC11.1* and *GmaVDAC1.1*/*GsoVDAC1.1*) in clade II, 16 genes and two pairs of orthologs (*GmaVDAC17.1*/*GsoVDAC17.1* and *GmaVDAC13.2*/*GsoVDAC13.2*) in clade III, 23 genes and four pairs of orthologs (*GmaVDAC6.1*/*GsoVDAC6.1*, *GmaVDAC4.1*/*GsoVDAC4.1*, *GmaVDAC17.2*/*GsoVDAC17.2*, and *GmaVDAC5.1*/*GsoVDAC5.1*) in clade IV, 15 genes and two ortholog pairs (*GmaVDAC18.1*/*GsoVDAC8.1* and *GmaVDAC8.1*/*GsoVDAC8.1*) in clade V, and 28 genes and four ortholog pairs (*GmaVDAC19.1*/*GsoVDAC19.1*, *GmaVDAC13.1*/*GsoVDAC13.1*, *GmaVDAC18.2*/*GsoVDAC18.2*, and *GmaVDAC9.1*/*GsoVDAC9.1*) in clade VI. In all clades, orthologous gene pairs in *G. max* and *G. soja* were found to be distributed on the same chromosome of the respective crop species. A tight clustering between *GmaVDAC* and *GsoVDAC* genes shows that they are more closely related to each other as compared to VDAC genes of any other species.

### 2.4. Conserved Motif and Gene Structure Analysis

**Figure 2:**
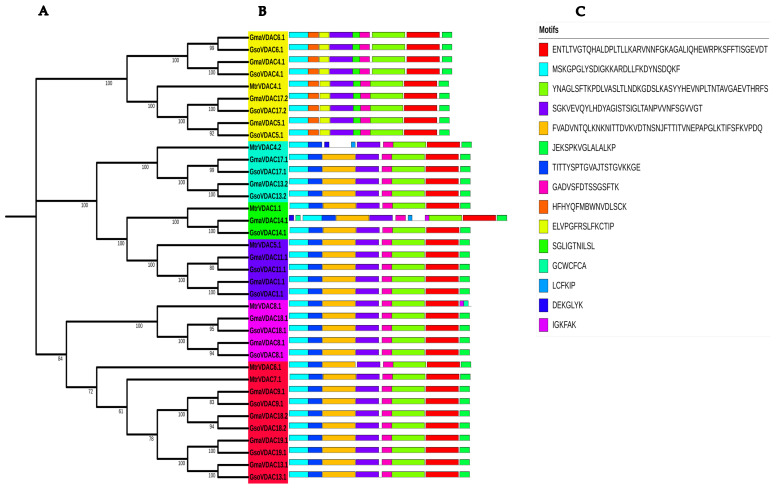
Phylogenetic tree and conserved motifs of VDAC proteins in *G. max*, *G. soja,* and *M. truncatula*. (**A**) There are six clades of the phylogenetic tree that are shown with six different colors (clade I = yellow; clade II = arctic blue; clade III = green; clade IV = dark blue; clade V = pink; and clade VI = red). (**B**) Conserved motifs of VDAC proteins are shown in different colors. (**C**) The amino acid sequence of each motif.

The presence of at least ten conserved motifs was searched out within *GmaVDAC*, *GsoVDAC*, and *MtrVDAC* proteins. Seven (1, 2, 3, 4, 5, 7, and 8) out of ten motifs were found to be highly conserved as they were present in all VDAC proteins selected for motif analysis (Figure 2).

**Figure 3:**
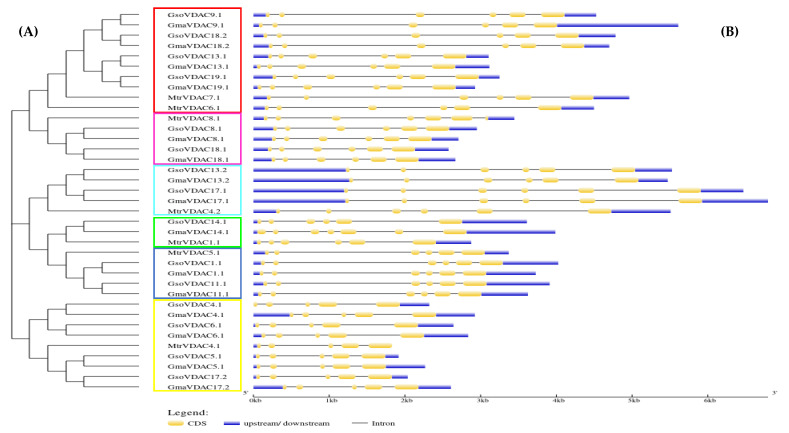
VDAC genes length, no. of exons and introns, and their order (**A**) in *G. max*, *G. soja,* and *M. truncatula* species along with the phylogenetic tree (**B**) classified into six clades with each shown with a specific color (clade I = yellow; clade II = arctic blue; clade III = green; clade IV = dark blue; clade V = pink and clade VI = red).

The diversity in the gene structures of *G. max*, *G. soja*, and *M. truncatula* was explored by drawing the exon–intron structure along with a phylogenetic tree, which revealed a small variation in the numbers of exons (5–6) of all VDAC genes (Figure 3). The genes of clade I possessed five exons whereas genes of other five clades consisted of six exons in each. However, it was found that the length and position of introns and exons were more similar within a clade as compared to that among the clades. This shows the conservation of gene structures in each clade.

### 2.5. Identification of Cis-Acting Regulatory Elements (CREs) in Promoters of GmaVDAC Genes

CREs are specific motifs involved in regulating the transcription of genes by binding to certain transcription factors [5]. The sequence 2 kb upstream from the promoter region of each *GmaVDAC* gene was analyzed for the identification of putative CREs. A total of 74 different *cis*-acting elements were found to be randomly distributed in promoters of all *GmaVDAC* genes. Based on their functions, these were divided into six categories relating to abiotic stress (17 elements), biotic stress (seven elements), growth and development (10 elements), phytohormones (13 elements), light responsiveness (20 elements), and transcription (six elements). A single element, i.e., the CTAG-motif, could not be placed in any category due to its unknown function.

**Figure 4:**
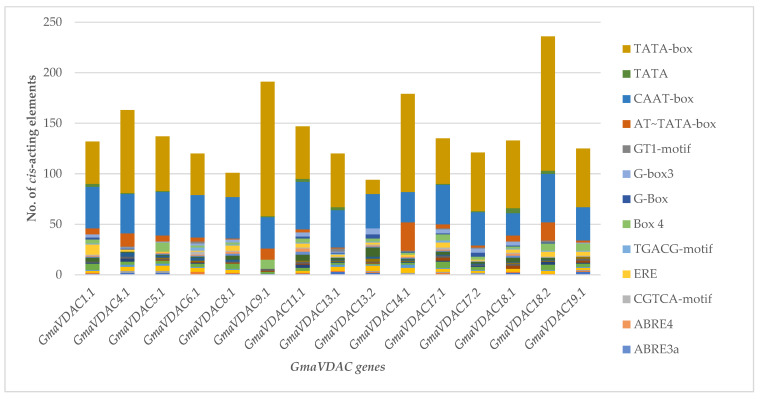
Cis-regulatory elements identified in promoters of 15 *GmaVDAC* genes: transcription-related elements (TATA-box, TATA, CAAT-box, and AT~TATA-box), light-responsive elements (GT1-motif, G-box 3, G-box, Box 4), hormone-responsive elements (TGACG-motif, ERE, CGTCA-motif, ABRE 4, ABRE 3a, and ABRE), and growth- and development-related elements (MYB-like sequence, Myb-binding site, and AAGAA-motif).

The most commonly found CREs in our studies included MYB (water responsive), MYC (drought responsive), ARE (anoxic responsive), WUN-motif (wound and pathogen responsive), ERE (ethylene responsive), ABRE (Abscisic acid responsive), Box 4 (light responsive), and G-box (light responsive). Similarly, the AT~TATA-box element (involved in transcription regulation) was found in all genes except *GmaVDAC13.2*. Two *cis*-elements, *viz*. CAAT-box (promoter or enhancer region) and TATA-box (promoter or enhancer region), were the most abundantly present in all *GmaVDAC* genes. Bearing in mind the major functions of *GmaVDAC* cis-acting elements, it can be predicted that the VDAC gene family plays a critical role in regulating abiotic stress in *G. max*. For better understanding, a graph (Figure 4) was drawn among 17 elements that were more than ten in number in all *GmaVDAC* genes.

### 2.6. Gene Duplication Events and Divergence Rate

The *G. max* genome has experienced two events of whole-genome duplication (WGD) and one event of whole-genome triplication (WGT), which has led to the presence of numerous duplicated gene copies. Approximately 75% of the soybean genome possesses multiple copies of genes [19]. Therefore, the genome duplication has resulted in the accumulation of triplet, tetrad, or paired homologous regions, leading to higher sequence similarity. The gene duplication analysis of 15 *GmaVDAC* genes was conducted to explore the diversity among gene sequences. The results of analysis show that the expansion of the VDAC gene family in soybean has been primarily driven by segmental duplications. In this study, seven segmental duplications were identified: *GmaVDAC1.1*/*GmaVDAC11.1*, *GmaVDAC4.1*/*GmaVDAC6.1*, *GmaVDAC5.1*/*GmaVDAC17.2*, *GmaVDAC8.1*/*GmaVDAC18.1*, *GmaVDAC9.1*/*GmaVDAC18.2*, *GmaVDAC13.1*/*GmaVDAC19.1*, and *GmaVDAC13.2*/*GmaVDAC17.1*.

The parameters such as Ka, Ks, and Ka/Ks values are used to assess the type of selection pressure that has influenced the codons. Generally, a Ka/Ks ratio greater than 1 signifies positive or Darwinian selection, while a Ka/Ks ratio less than 1 indicates negative, purifying, or stabilizing selection. However, a Ka/Ks ratio equal to 1 suggests neutral selection. With a Ka/Ks ratio ranging from 0.06 to 0.23, purifying selection was observed in all seven duplication events. Furthermore, the approximate divergence time of gene duplication events was calculated, revealing that these events likely occurred between 5.16 and 12.51 million years ago (MYA). The complete list of *GmaVDAC* duplicates along with Ka, Ks, Ka/Ks values, the estimated time of divergence, and duplicate and selection types are provided in Table 1.

### 2.7. Gene Positions and Chromosomal Chart

**Figure 5:**
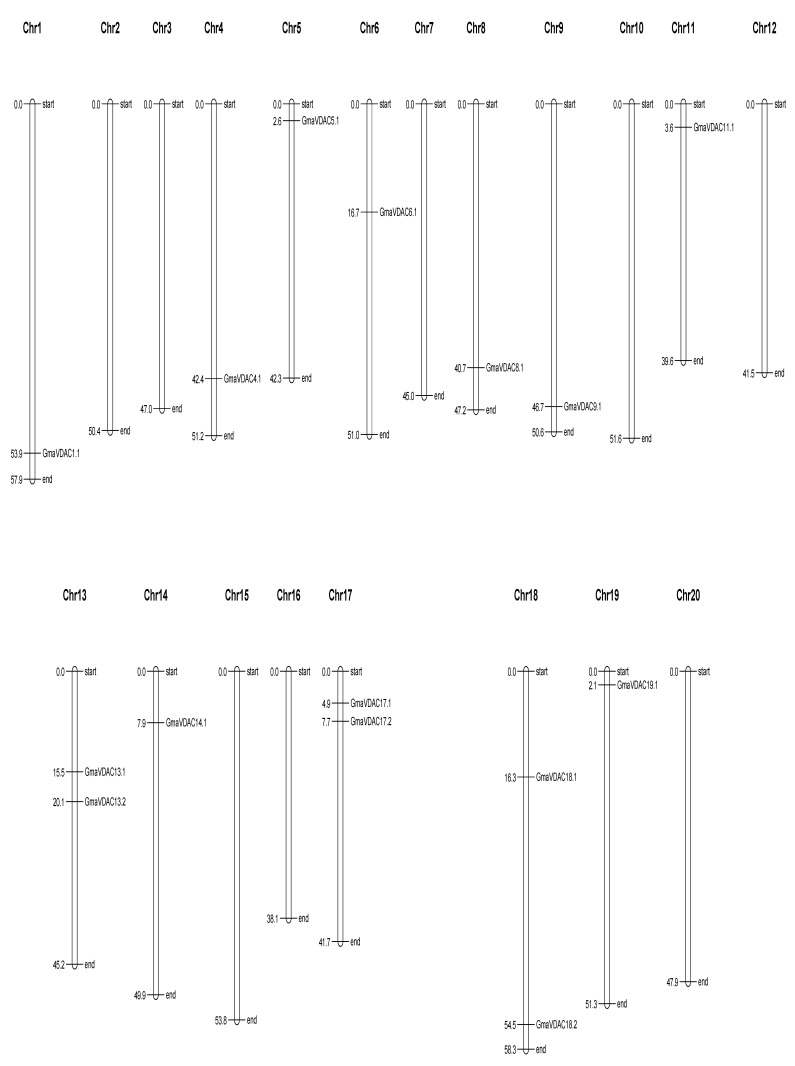
Locations of *GmaVDAC* genes on chromosomes. The positions are given in megabases on the left side while the corresponding gene names are given on the right side. No VDAC genes were found on chromosomes 2, 3, 7, 10, 12, 15, 16, and 20.

The localization pattern of VDAC genes in *Glycine max* was examined and it was found that *GmaVDAC* genes were unevenly distributed among chromosomes. These genes were found on 12 of the 20 pairs of chromosomes (Figure 5). Chromosomes 1, 4, 5, 6, 8, 9, 11, 14, and 19 each carried one VDAC gene, while chromosomes 13, 17, and 18 contained two VDAC genes. The remaining chromosomes did not possess any VDAC genes.

### 2.8. Synteny Analysis

**Figure 6:**
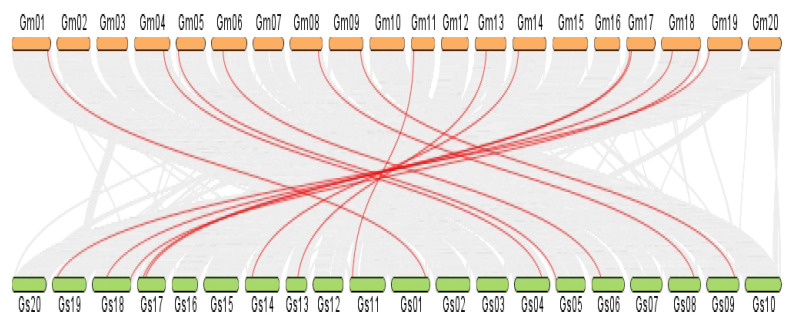
Syntenic plot made between chromosomes of *G. max* (orange color) and *G. soja* (green color) showing orthologous VDAC genes in red lines.

To identify orthologous genes, a dual synteny plot was constructed between cultivated soybean (*G. max*) and wild soybean (*G. soja*). We found 14 homologous VDAC gene pairs for 15 *GmaVDAC* genes and there was a single VDAC gene on chromosome 13 of *G. max* for which no homolog was identified in *G. soja*. In addition, it was found that all homologs in two species were present on corresponding chromosomes (Figure 6). This shows that they remain highly conserved during the evolutionary process.

### 2.9. Expression Pattern Analysis of GmaVDAC Genes

**Figure 7:**
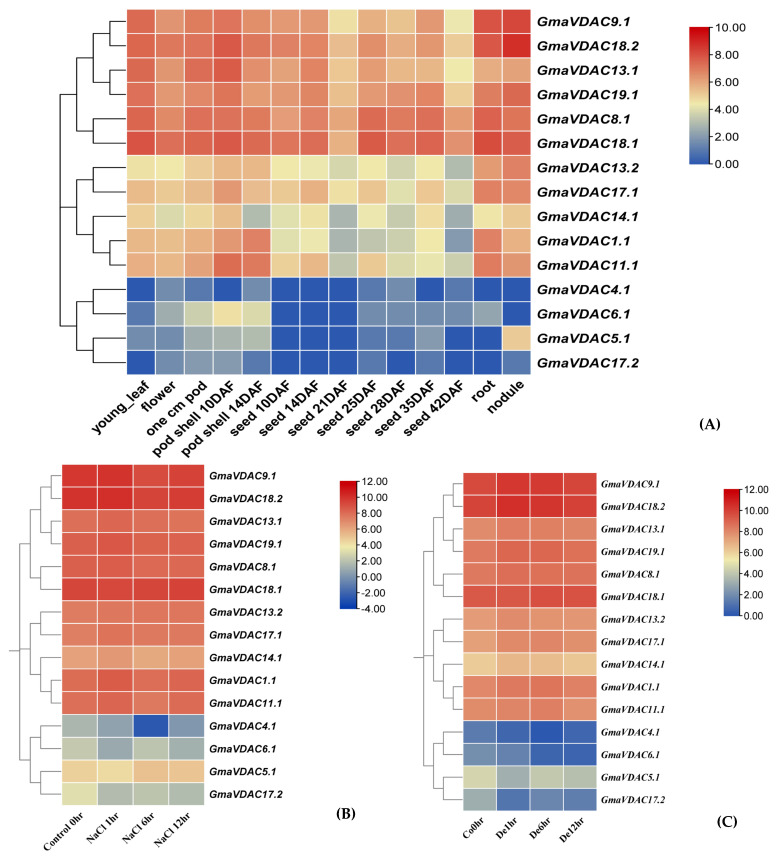
Expression pattern of *GmaVDAC* genes obtained through RNA-seq data. (**A**) Differentially expressed genes in 14 tissues of soybean plants (vegetative and reproductive tissues). (**B**) Expression under salt conditions (1 h, 6 h, and 12 h). (**C**) Expression under drought conditions (1 h, 6 h, and 12 h). Intensity of color shows the level of expression (the red color shows the highest expression, whereas the blue color shows the lowest expression).

The normalized RNA expression data of *GmaVDAC* genes in fourteen plant parts was retrieved from SoyBase and log_2_ transformed. The expression pattern of *GmaVDAC* genes highly varied among fourteen parts (Figure 7A). Four genes viz. *GmaVDAC4.1*, *GmaVDAC5.1*, *GmaVDAC6.1*, and *GmaVDAC17.2* showed the lowest expression in all studied plant parts. However, the genes *GmaVDAC8.*1, *GmaVDAC18.1*, *GmaVDAC9.1*, *GmaVDAC18.2*, and *GmaVDAC19.1* were expressed relatively higher in all tissues. In addition, it was found that the *GmaVDAC18.2* gene showed the highest expression in nodules of plants.

Similarly, the expression profiles of *GmaVDAC* genes under salt and drought stress conditions were also analyzed (Figure 7B and 7C), respectively. After one hour of salt and drought stress exposure, the genes *GmaVDAC1.1*, *GmaVDAC8.1*, *GmaVDAC9.1*, *GmaVDAC11.1*, *GmaVDAC13.1*, *GmaVDAC13.2*, *GmaVDAC14.1*, *GmaVDAC17.1*, *GmaVDAC18.2*, and *GmaVDAC19.1* were found to be upregulated. The expression of four genes, viz. *GmaVDAC4.1*, *GmaVDAC5.1*, *GmaVDAC6.1*, and *GmaVDAC17.2*, was found to be negligible compared to other VDAC genes under both conditions. After 6 and 12 h of salt and drought stress, it was noticed that most of the genes downregulated except for *GmaVDAC18.1*, *GmaVDAC18.2*, and *GmaVDAC9.1*, which expressed upregulation.

### 2.10. Predicted Functions of GmaVDAC Genes

**Figure 8:**
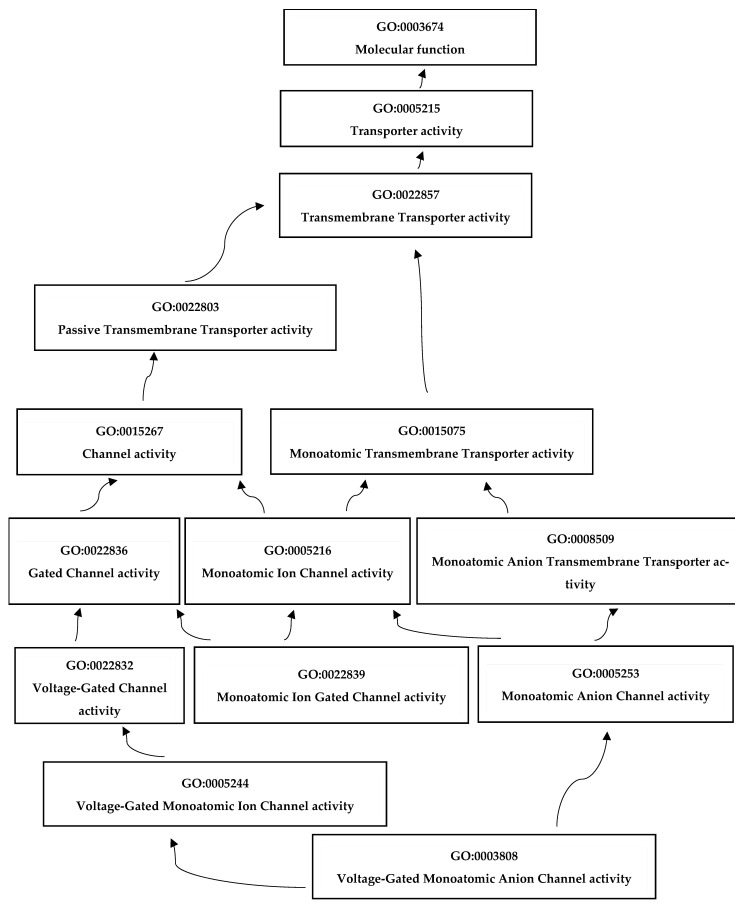
We present a chart illustrating the molecular function annotations of the *GmaVDAC* gene family in *G. max*, as determined through Gene Ontology (GO) analysis using Blast2GO. Key molecular functions include voltage-gated anion channel activity (GO:0008308) and porin activity (GO:0015288), providing insights into the functional roles of these genes.

Research in functional genomics relies on the annotation of transcriptomic sequences, and one of the key methods for this annotation is gene ontology (GO) analysis. In our study, we performed GO analysis of the VDAC gene family in *G. max*, which provided valuable insights into the functional characteristics and roles of *GmaVDAC* genes. The four GO terms obtained were as follows: (i) biological processes including, inorganic anion transport (GO:0015698) and mono-atomic anion transmembrane transport (GO:0098656); (ii) the molecular function of voltage gated anion channel activity (GO:0008308), but a closely similar molecular function annotation in porin activity (GO:0015288) was also obtained from the STRING database; and (iii) cellular localization in mitochondrial the outer membrane (GO:0005741). Using Blast2GO, we created a chart showing molecular functions (Figure 8).

### 2.11. Protein Interactions and microRNA Targets

**Figure 9:**
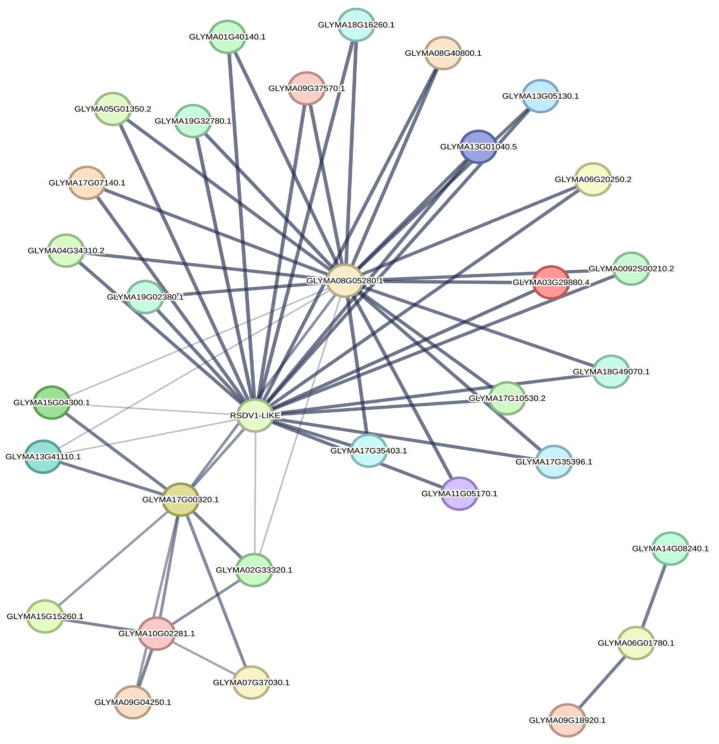
Interaction network of proteins involved in porin activity in *G. max* (the thickness of lines indicates the strength of interactions among them).

The gene ontology predicted the role of VDAC proteins in porin activity (GO:0015288). The string database was explored to determine interactions among proteins involved in porin activity. The search was performed with the query ‘GO:0015288’. The analysis was conducted by setting a medium confidence level (0.400) and none of the interactors involved in the 1st and 2nd subshells. The analysis revealed 32 interacting proteins along with 52 edges or interactions among them (Figure 9). Interestingly, no interaction was seen among members of the GmaVDAC protein family; however, we found an interaction between each GmaVDAC protein and two mitochondrial import receptors (RSVD1 like and Glyma08G05280.1) that may indirectly activate VDAC proteins during the imbalance of ions. In addition, three interacting proteins, i.e., Glyma9G18920.1, Glyma6G01780.1, and Glyma14G08240.1, were found outside the network, indicating no interaction with GmaVDAC proteins. But these are also involved in regulating porin activity. These interactions suggest that VDAC proteins play an important role in regulating the traffic of substances, especially anions, across mitochondrial membrane.

**Figure 10:**
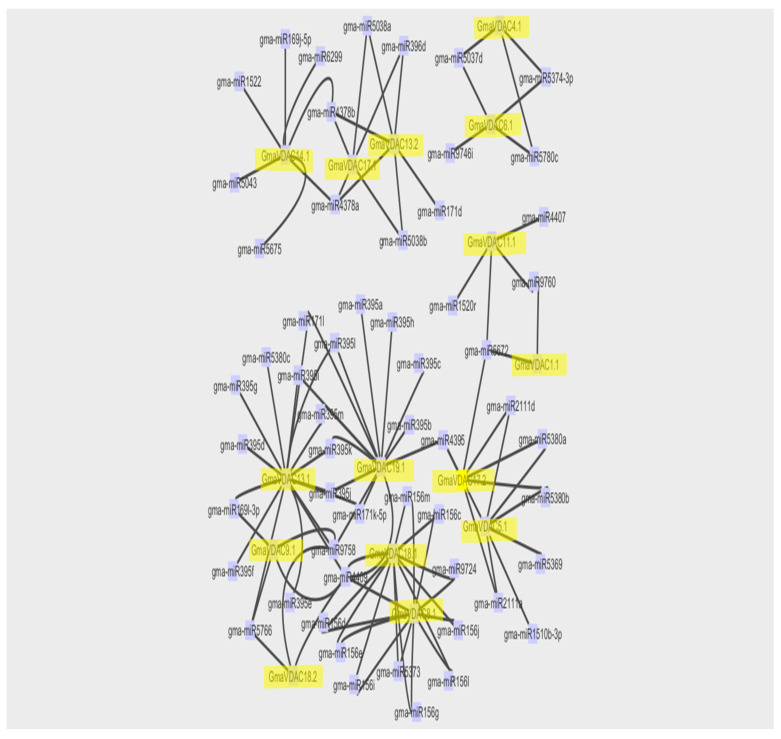
The schematic representation of the regulatory network relationships between putative miRNAs and their targeted soybean VDAC genes.

MicroRNAs (miRNAs) play a crucial role in regulating gene expression by either degrading mRNAs or inhibiting protein translation [20]. However, the transcriptional regulation of miRNA genes remains largely unknown. Therefore, we conducted a systematic analysis to explore potential miRNAs targeting the soybean VDAC gene family. This analysis aimed to provide insights into the regulatory mechanism of *GmaVDAC* genes. Our findings revealed 103 miRNAs targeting 15 *GmaVDAC* genes. Each gene was associated with 2 to 16 miRNAs, and a relationship network was constructed (Figure 10). Noticeably, *GmaVDAC13.1* and *GmaVDAC19.1* were the most targeted genes in soybean, with 16 and 14 predicted miRNA targets, respectively. Additionally, *GmaVDAC1.1* was targeted by two miRNAs (gma-miR5672 and gma-miR9760). Furthermore, we observed that two genes (*GmaVDAC4.1* and *GmaVDAC18.2*) were targeted by three miRNAs, three genes (*GmaVDAC6.1*, *GmaVDAC9.1*, and *GmaVDAC11.1*) were targeted by four miRNAs, two genes (*GmaVDAC13.2* and *GmaVDAC17.2*) were targeted by six miRNAs, two genes (*GmaVDAC5.1* and *GmaVDAC14.1*), and two other genes (*GmaVDAC8.1* and *GmaVDAC18.1*) were targeted by 11 miRNAs. Similarly, five miRNAs were found in association with the *GmaVDAC17.1* gene.

## 3. Discussion

VDAC proteins serve as a crucial pathway for transporting various substances, ranging from ions to large biomolecules. The expression of VDAC genes is influenced by environmental stress. In the present study, an extensive analysis was conducted on the VDAC gene family in 13 legume species (including cultivated and wild soybeans) along with *Arabidopsis thaliana*.

A total of 111 VDAC genes were identified across all studied plant species including 15 genes in each of two soybean species, i.e., G. *max* and *G. soja*. This shows a high structural and functional similarity among VDAC genes in soybeans. However, their varying numbers from four (*C. cajan*) to seventeen (*A. hypogaea*) across other legumes indicates their diversity in sequence structure and functional roles. In a previous study [21], five VDAC genes were reported in *G. max* that contradicted our results. The present study identified five VDAC genes in *Arabidopsis thaliana*; however, four genes were previously reported in this species [22]. Similarly, five genes were identified in *Medicago truncatula* and the same number of genes were also reported [15].

The comparative phylogenetic tree exhibited a tight clustering between *GmaVDAC* and *GsoVDAC* genes. This shows the highest sequence similarity among VDAC genes of these two species compared to those of any other species studied. This close similarity is possibly due to a shared ancestral origin. *G. max* and *G. soja* shared an equal number of VDAC genes in each clade, suggesting that these gene sequences remained conserved during the domestication of cultivated soybean from wild species. Moreover, six clades were formed in the tree in present study. Similarly, the VDAC gene family was characterized in wheat and reported the same number of clades in a comparative phylogenetic tree [4].

The sub-cellular localization in the mitochondria of all VDAC protein members indicates their significance for the proper functioning of this organelle [23]. Their localization in mitochondria and small unknown vesicles was also reported in soybean [21]. A small variation in the number of introns and exons of VDAC genes of both cultivated and wild soybeans suggests that the genes remain conserved throughout the process of evolution due to their great functional importance. Seven highly conserved motifs in both *G. max* and *G. soja* species show a very close sequence similarity in VDAC proteins and the preservation of VDAC gene structures in both soybean species. Genes that share similar structures in both species also exhibit a similar composition of motifs. Most of the conserved *GmaVDAC* protein motifs (i.e., 1, 3, 4, 5, 7, 8, and 9) reported in this study were also described as conserved in wheat [4].

Gene duplications play a significant role in genome evolution and enhance the capacity of the genome to adapt to changing environments [24]. The variation in the number of VDAC genes among different legume species can be attributed to this force [25]. In *G. max* and *G. soja*, the higher number of VDAC genes compared to the model legume *M. truncatula* (which has 7 VDACs) is primarily due to segmental duplication. Specifically, seven segmental duplicates were identified in *GmaVDAC* genes. Both genes of each paralogous pair identified in *G. max* exhibited not less than 90% sequence similarity with each other. The divergence time of duplication events also varied in *GmaVDAC* genes. A higher value of Ka/Ks indicates a greater divergence in the duplication event [26]. In *G. max*, the segmental duplication events of VDAC genes occurred between 5.16 and 12.51 million years ago (Mya). These findings suggest that gene duplications in *G. max* occurred recently for this gene family. These results align closely with the findings of [27], who proposed that duplication events in the Glycine lineage occurred around 13 Mya. Though, the distribution of *GmaVDAC* and *GsoVDAC* genes on chromosomes was uneven, their corresponding genomic positions in both species had more than 90% sequence similarities.

VDAC proteins act as gatekeepers in the mitochondrial outer membrane and thus contribute to defense against abiotic stress, especially drought and salt stress, by regulating the movement of ions between the cytoplasm and mitochondria. Previous studies have demonstrated the roles of VDAC genes in conferring tolerance to drought and salt stress [4]. Various *cis*-regulatory elements were identified in the promoter region of *GmaVDAC* genes, which were mainly related to growth and development, abiotic and biotic stress tolerance, transcription regulation, and light responsiveness. The varied number of different *cis* elements is an indication of the diverse roles of *GmaVDAC* genes. The presence of certain elements like ARE, as-1, Box-4, Myb, MYC, GT1-motif, WUN-motif, and STRE indicates that these genes respond to drought, salinity, cold, and hormonal conditions as was previously described by [4,15,16,17].

To validate the expression patterns of VDAC genes at various developmental stages, we examined their expression using RNA-seq data of *G. max*. These findings highlighted the tissue-specific expression of VDAC genes in response to developmental cues. The contiguous expression of VDAC genes was reported in all plant parts including root nodules in *L. japonicus* [21]. Similarly, the overexpression of *GmaVDAC9.1*, *GmaVDAC18.1*, and *GmaVDAC18.2* genes also showed their potential role in drought and salt stress tolerance as evidenced in wheat [4].

The gene ontology results confirmed the role of *GmaVDAC* proteins in voltage-gated anion channel activity or porin activity. These molecular functions were also described by many researchers [3,7]. The protein interaction network revealed that there was no interaction among *GmaVDAC* protein family members. This shows that the expression of one protein does not affect the expression of any other *GmaVDAC* protein. Nonetheless, their interactions with two mitochondrial import receptors suggest that the activation of VDAC proteins may be regulated by these receptor proteins. In addition, 103 miRNA targets were predicted in 15 *GmaVDAC* genes. *GmaVDAC13.1* and *GmaVDAC19.1* genes were found to be targeted by 16 and 14 miRNAs, respectively.

## 4. Materials and Methods

### 4.1. Identification of VDAC Genes and Retrieval of Sequences

In this study, we searched out and retrieved the genomic, transcriptomic, and proteomic sequences of VDAC genes from the genomes of 13 legume species and a model plant species, *Arabidopsis thaliana* (Table 2) [2].

### 4.2. Conserved Domain, Subcellular Localization, and Physico-Chemical Properties

We found the hidden Markov model (HMM) of VDAC proteins from the Pfam database (https://pfam.xfam.org/; accessed on 21 July 2022) and confirmed the presence of the PF01459 domain in each VDAC protein from the conserved domain database (CDD) of the NCBI (https://www.ncbi.nlm.nih.gov/cdd; accessed on 21 July 2022) [28].

For subcellular location, we used WegoLoc (https://www.btool.org/WegoLoc; accessed on 25 July 2022) [29]. Moreover, the physico-chemical properties of VDAC proteins were determined using the ProtParam (https://web.expasy.org/protparam/; accessed on 25 July 2022) tool of the Expasy website [30].

### 4.3. Multiple Sequence Alignment (MSA) and Phylogenetic Relationship

We conducted multiple sequence alignment (MSA) of all VDAC proteomic sequences of selected plant species using the ClustalW algorithm of MEGA11 software. Further, we performed the phylogenetic analysis and constructed a comparative phylogenetic tree in MEGA11 [31]. Thereafter, the phylogenetic tree was edited and modified using the iTOL website to make it easier to understand (https://itol.embl.de/; accessed on 9 August 2022).

### 4.4. Gene Structure Prediction and Motif Analysis

The Gene Structure Display Server (GSDS) (https://gsds.gao-lab.org/; accessed on 22 August 2022) was employed to predict the structure of VDAC genes in three species, *viz. G. max*, *G. soja*, and *M. truncatula* [32]. For conserved motifs, MEME tool (https://meme-suite.org/meme/tools/meme; accessed on 22 August 2022) was used by considering at least ten motifs for each VDAC protein with a motif width ranging from 6 to 50 [33].

### 4.5. Cis-Regulatory Elements (CREs) and Chromosomal Distribution

In *G. max*, we predicted the *cis*-regulatory elements (CREs) in the 2000 bp upstream promoter region of the gene transcript by using the CARE searching tool of the PlantCARE database (https://bioinformatics.psb.ugent.be/webtools/plantcare/html/; accessed on 27 August 2022) [34]. To show gene positions, we used VDAC genomic sequences of *G. max* to draw a physical map in MapChart (v1) software [35].

### 4.6. Gene Duplications and Syntenic Analysis

Genes with 90% or more similarity in sequences were considered as duplicated genes. Generally, there are two types of duplications, i.e., tandem duplications (if genes are separated by fewer than or up to five genes) and segmental duplications (if genes are scattered on different chromosomes or are separated by more than five genes) [2].

For this purpose, we employed MCScanX (Multiple Collinearity Scan Toolkit v2) with default parameters [36]. After sequence alignment, DnaSP v5.10.01 software was used to perform synonymous (Ks) analysis to calculate Ks values and non-synonymous (Ka) analysis was used to calculate Ka values of substitution [37]. The Ka/Ks ratio was calculated from the SNAP web-based tool to further analyze the selection mode and selection pressure during evolutionary process. The estimated time of the divergence event was calculated by using the formula T = Ks/2λ where λ = 6.1 × 10^−9^ [27]. Similarly, we constructed syntenic plots to show the syntenic relationship among VDAC paralogs in *G. max* by using TBtools (v1.0692) [38].

### 4.7. Protein Interaction Network and microRNA Target Prediction

The possible protein–protein interaction network and microRNA targets of *GmaVDAC* genes were predicted by using the STRING database (https://string-db.org/; accessed on 12 January 2023) and psRNATarget (https://plantgrn.noble.org/psRNATarget/ accessed on 15 January 2023), respectively [39,40]. Both interaction networks were drawn using Cytoscape v3.9.1 [41].

### 4.8. Gene Ontology Analysis

To describe the functional annotation of *GmaVDAC* genes, peptide sequences of GmaVDAC proteins were uploaded on Blast2GO (https://www.blast2go.com/; accessed on 7 February 2023) and the results were visualized using BiNGO (https://apps.cytoscape.org/apps/bingo; accessed on 8 February 2023) [42]. The GO analysis provides insights into the cellular components, molecular functions, and biological processes of the concerned genes [20].

### 4.9. Expression Pattern of GmaVDAC Genes

The expression analysis of *GmaVDAC* genes in 14 different tissues of the soybean plant was studied using transcriptomic data previously submitted to the SoyBase website (https://www.soybase.org/soyseq/; accessed on 12 February 2023) by [43]. The normalized RNA-seq data of different growth stages was downloaded, log_2_ transformed, and the results were displayed in the form of a heatmap using TBtools [38].

Moreover, we conducted an in-depth examination of the *GmaVDAC* gene expression analysis regarding drought and salt stress. We achieved this by analyzing publicly accessible RNA-seq data that were available on the Gene Expression Omnibus (GEO) database, specifically under the accession number GSE57252 of *G. max* [44].

## 5. Conclusions

To date, no comprehensive study of the VDAC gene family has been conducted in soybeans. Therefore, this research provides the first systematic and extensive investigation of VDAC genes in soybean. A total of 111 VDAC genes were identified and analyzed for phylogenetic studies in fourteen crop species including *G. max* and *G. soja*. The conserved gene structures and protein domains indicate their importance for the proper functioning of a cell. The expansion of VDAC genes in *G. max* occurred through segmental duplication and purifying selection. Their expression patterns varied among plant tissues, with the highest expression in root nodules. Similarly, the transcriptomic profile revealed the upregulation of *GmaVDAC* genes under drought and salt stress conditions. The gene annotations and protein interactions suggested their role in voltage-gated anion channel activity. Overall, this study provides valuable insights into the functions of VDAC proteins that can be further explored to harness their potential for drought and salt tolerance in soybeans.

## Figures and Tables

**Table 1:** List of *GmaVDAC* duplicates including their type, Ka, Ks and Ka/Ks values, time of divergence and selection type.

Duplicated Pair	Ka	Ks	Ka/Ks	Duplicate Type	Selection Type	T (Mya)
*GmaVDAC1.1*/*GmaVDAC11.1*	0.01	0.07	0.18	Segmental	Purifying	6.10
*GmaVDAC4.1*/*GmaVDAC6.1*	0.03	0.14	0.21	Segmental	Purifying	11.39
*GmaVDAC5.1*/*GmaVDAC17.2*	0.02	0.09	0.23	Segmental	Purifying	7.02
*GmaVDAC8.1*/*GmaVDAC18.1*	0.01	0.14	0.06	Segmental	Purifying	11.31
*GmaVDAC9.1*/*GmaVDAC18.2*	0.005	0.06	0.08	Segmental	Purifying	5.16
*GmaVDAC13.1*/*GmaVDAC19.1*	0.02	0.15	0.12	Segmental	Purifying	12.51
*GmaVDAC13.2*/*GmaVDAC17.1*	0.02	0.11	0.18	Segmental	Purifying	8.75

**Table 2:** List of crop species for phylogenetic studies and database links for retrieval of VDAC genes sequences.

Sr. No.	Crop	Botanical name	Database link
1.	Adzuki bean	*Vigna angularis*	https://legacy.legumeinfo.org/ (accessed on 17 June 2022)
2.	Arabidopsis	*Arabidopsis thaliana*	https://www.arabidopsis.org/ (accessed on 17 June, 2022)
3.	Barrel clover	*Medicago truncatula*	https://phytozome-next.jgi.doe.gov/ (accessed on 20 June 2022)
4.	Birdsfoot trefoil	*Lotus japonicus*	https://phytozome-next.jgi.doe.gov/ (accessed on 20 June 2022)
5.	Chickpea	*Cicer arietinum*	https://phytozome-next.jgi.doe.gov/ (accessed on 21 June 2022)
6.	Common bean	*Phaseolus vulgaris*	https://phytozome-next.jgi.doe.gov/ (accessed on 21 June 2022)
7.	Cowpea	*Vigna unguiculata*	https://phytozome-next.jgi.doe.gov/ (accessed on 21 June 2022)
8.	Cultivated soybean	*Glycine max*	https://www.soybase.org/ (accessed on 21 June 2022)
9.	Mungbean	*Vigna radiata*	https://legacy.legumeinfo.org/ (accessed on 23 June 2022)
10.	Narrow leaf lupin	*Lupinus angustifolius*	https://legacy.legumeinfo.org/ (accessed on 23 June 2022)
11.	Pea	*Pisum sativum*	https://legacy.legumeinfo.org/ (accessed on 23 June 2022)
12.	Peanut	*Arachis hypogaea*	https://phytozome-next.jgi.doe.gov/ (accessed on 24 June 2022)
13.	Pigeon pea	*Cajanus cajan*	https://legacy.legumeinfo.org/ (accessed on 24 June 2022)
14.	Wild soybean	*Glycine soja*	https://www.soybase.org/ (accessed on 24 June 2022)

## Data Availability

Data is contained within the article and Supplementary Materials.

## Supplementary Materials

The following supporting information can be downloaded at https://www.mdpi.com/article/10.3390/plants14142101/s1: Table S1: List of identified VDAC genes in 14 crop species including cultivated and wild soybeans with genomic and proteomic features.
