# Supplementary material for: Genome-Wide Characterization of VDAC Gene Family in Soybean (Glycine max L.) and In Silico Expression Profiling in Response to Drought and Salt Stress
Source: Plants (Basel). 2025 Jul 8;14(14):2101. doi: 10.3390/plants14142101 (PMC12300895; doi:10.3390/plants14142101)
Supplement: Supplementary file 1 [file plants-14-02101-s001.zip › plants-3703052-supplementary.pdf]

Table S1. List of identified VDAC genes in 14 crop species including cultivated and wild soybeans with genomic and proteomic features

		Gene features								Protein features						
**Species**	**Sr. No.**	**Gene ID**	**Renamed ID**	**Chr. No.**	**Direction**	**Start (bp)**	**End (bp)**	**Length (bp)**	**CDS (bp)**	**Pfam**	**Domain**	**AA**	**MW (kDa)**	**pI**	**GRAVY**	**Localization**
A. hypogaea	1	A.hypogaea v1.0.gnm1.ann1.8J5KLD.Arahy.01	*AhyVDAC1.1*	Chr 1	reverse	96195685	96202928	7243	1962	PF01459	Porin – 3	653	73308	8.6	-0.201	Mitochondria
	2	A.hypogaea v1.0.gnm1.ann1.E7ZXJI.Arahy.01	*AhyVDAC1.2*	Chr 1	forward	111762125	111767460	5335	735	PF01459	Porin – 3	244	26341	6.29	0.043	Mitochondria
	3	A.hypogaea v1.0.gnm1.ann1.3VS4JG.Arahy.03	*AhyVDAC3.1*	Chr 3	reverse	134288696	134291436	2740	831	PF01459	Porin – 3	276	29689	6.38	-0.171	Mitochondria
	4	A.hypogaea v1.0.gnm1.ann1.SIN6AN.Arahy.04	*AhyVDAC4.1*	Chr 4	reverse	122067025	122070421	3396	831	PF01459	Porin – 3	276	29647	7.85	-0.219	Mitochondria
	5	A.hypogaea v1.0.gnm1.ann1.F7SZPJ.Arahy.05	*AhyVDAC5.1*	Chr 5	reverse	6910686	6913422	2736	831	PF01459	Porin – 3	276	29372	9.13	-0.146	Mitochondria
	6	A.hypogaea v1.0.gnm1.ann1.52N2TQ.Arahy.05	*AhyVDAC5.2*	Chr 5	reverse	18392118	18395147	3029	831	PF01459	Porin – 3	276	29729	8.86	-0.212	Mitochondria
	7	A.hypogaea v1.0.gnm1.ann1.DP5746.Arahy.05	*AhyVDAC5.3*	Chr 5	reverse	108425355	108427324	1969	774	PF01459	Porin – 3	257	28087	5.46	-0.045	Mitochondria
	8	A.hypogaea v1.0.gnm1.ann1.5JCW6G.Arahy.0 6	*AhyVDAC6.1*	Chr 6	reverse	112705632	112708374	2742	840	PF01459	Porin – 3	279	29763	9.04	-0.077	Mitochondria
	9	A.hypogaea v1.0.gnm1.ann1.YE2V2Z.Arahy.10	*AhyVDAC10.1*	Chr 10	reverse	107523346	107526873	3527	831	PF01459	Porin – 3	276	29840	8.53	-0.256	Mitochondria
	10	A.hypogaea v1.0.gnm1.ann1.CNIN65.Arahy.11	*AhyVDAC11.1*	Chr 11	reverse	121015443	121019840	4397	735	PF01459	Porin – 3	244	26355	6.58	0.041	Mitochondria
	11	A.hypogaea v1.0.gnm1.ann1.VJ3SQ3.Arahy.11	*AhyVDAC11.2*	Chr 11	forward	147892342	147896636	4294	834	PF01459	Porin – 3	277	29914	8.96	-0.161	Mitochondria
	12	A.hypogaea v1.0.gnm1.ann1.QM0EZ0.Arahy.1 5	*AhyVDAC15.1*	Chr 15	reverse	6843875	6846556	2681	831	PF01459	Porin – 3	276	29358	9.13	-0.147	Mitochondria
	13	A.hypogaea v1.0.gnm1.ann1.L6SVEW.Arahy.1 5	*AhyVDAC15.2*	Chr 15	forward	38044556	38047602	3046	831	PF01459	Porin – 3	276	29743	8.86	-0.211	Mitochondria
	14	A.hypogaea v1.0.gnm1.ann1.PB3V0K.Arahy.15	*AhyVDAC15.3*	Chr 15	forward	127190669	127193294	2625	774	PF01459	Porin – 3	257	28074	5.3	-0.032	Mitochondria
A. thaliana	1	AT3G01280	*AtVDAC3.1*	Chr3	forward	85600	87858	2258	831	PF01459	Porin – 3	276	29425	8.77	-0.114	Mitochondria
	2	AT3G49920	*AtVDAC3.2*	Chr3	reverse	18505835	18507835	2000	681	PF01459	Porin – 3	226	24631	9.19	-0.222	Mitochondria
	3	AT5G15090	*AtVDAC5.1*	Chr5	reverse	4888963	4891603	2640	825	PF01459	Porin – 3	274	29211	7.84	-0.183	Mitochondria
	4	AT5G57490	*AtVDAC5.2*	Chr5	reverse	23283679	23285934	2255	825	PF01459	Porin – 3	274	29505	9.23	-0.166	Mitochondria
	5	AT5G67500	*AtVDAC5.3*	Chr5	forward	26935142	26937483	2341	912	PF01459	Porin – 3	303	32905	7.12	-0.217	Mitochondria
C. arietinum	1	Ca_10539	*CarVDAC2.1*	Chr 2	reverse	3104538	3108355	3817	831	PF01459	Porin – 3	276	29907	8.86	-0.251	Mitochondria
	2	Ca_20683	*carVDAC2.2*	Chr 2	reverse	1315148	1319023	3875	831	PF01459	Porin – 3	276	29513	9	-0.175	Mitochondria
	3	Ca_18368	*CarVDAC4.1*	Chr 4	forward	45917851	45919953	2102	834	PF01459	Porin – 3	277	29922	9.21	-0.22	Mitochondria

	4	Ca_03253	*CarVDAC7.1*	Chr 7	forward	2167778	2172670	4892	945	PF01459	Porin – 3	314	34047	9.01	-0.252	Mitochondria
	5	Ca_15337	*CarVDAC7.2*	Chr 7	forward	8774847	8776568	1721	732	PF01459	Porin – 3	243	26683	8.32	-0.002	Mitochondria
	6	Ca_02221	*CarVDAC8.1*	Chr 8	reverse	3551326	3554655	3329	831	PF01459	Porin – 3	276	29531	9.4	-0.151	Mitochondria
C. cajan	1	cajca.C.cajan_08221.1	*CcaVDAC3.1*	Chr 3		1757633	1761518	3885	831	PF01459	Porin – 3	276	29709	8.87	-0.208	Mitochondria
	2	cajca.C.cajan_14403.1	*CcaVDAC10.1*	Chr 10		12217861	12220102	2241	1200	PF01459	Porin – 3	399	Undefined	Undefined	-0.093	Mitochondria
	3	cajca.C.cajan_01675	*CcaVDAC11.1*	Chr 11		18325066	18327094	2028	738	PF01459	Porin – 3	245	26684	6.18	-0.012	Mitochondria
	4	cajca.C.cajan_03638	*CcaVDAC11.2*	Chr 11		40152226	40156427	4201	786	PF01459	Porin – 3	261	28361	8.39	-0.2	Mitochondria
G. max	1	Glyma.01G193700	*GmaVDAC1.1*	Chr1	forward	53939379	53943107	3728	831	PF01459	Porin – 3	276	29116	9.11	-0.098	Mitochondria
	2	Glyma.04G174300	*GmaVDAC4.1*	Chr4	forward	42400051	42402975	2924	747	PF01459	Porin – 3	248	27064	7.73	-0.17	Mitochondria
	3	Glyma.05G029700	*GmaVDAC5.1*	Chr5	forward	2548885	2551152	2267	738	PF01459	Porin – 3	245	26545	5.96	0.019	Mitochondria
	4	Glyma.06G190400	*GmaVDAC6.1*	Chr6	reverse	16664371	16667207	2836	747	PF01459	Porin – 3	248	26783	8.81	-0.16	Mitochondria
	5	Glyma.08G296700	*GmaVDAC8.1*	Chr8	reverse	40679482	40682190	2708	831	PF01459	Porin – 3	276	29804	7.07	-0.237	Mitochondria
	6	Glyma.09G240600	*GmaVDAC9.1*	Chr9	forward	46684151	46689761	5610	831	PF01459	Porin – 3	276	29756	8.57	-0.188	Mitochondria
	7	Glyma.11G048200	*GmaVDAC11.1*	Chr11	reverse	3585499	3589124	3625	831	PF01459	Porin – 3	276	29208	9.29	-0.09	Mitochondria
	8	Glyma.13G064900	*GmaVDAC13.1*	Chr13	forward	15504323	15507439	3116	831	PF01459	Porin – 3	276	29763	9.05	-0.241	Mitochondria
	9	Glyma.13G096000	*GmaVDAC13.2*	Chr13	forward	20114152	20119623	5471	834	PF01459	Porin – 3	277	29757	8.66	-0.159	Mitochondria
	10	Glyma.14G088800	*GmaVDAC14.1*	Chr14	reverse	7886806	7890793	3987	1002	PF01459	Porin – 3	333	35938	9.43	-0.107	Mitochondria
	11	Glyma.17G064100	*GmaVDAC17.1*	Chr17	reverse	4933090	4939885	6795	834	PF01459	Porin – 3	277	29961	8.99	-0.178	Mitochondria
	12	Glyma.17G097100	*GmaVDAC17.2*	Chr17	reverse	7650249	7652857	2608	738	PF01459	Porin – 3	245	26637	6.29	0.024	Mitochondria
	13	Glyma.18G125500	*GmaVDAC18.1*	Chr18	forward	16342518	16345185	2667	831	PF01459	Porin – 3	276	29789	7.07	-0.208	Mitochondria
	14	Glyma.18G255700	*GmaVDAC18.2*	Chr18	reverse	54462002	54466701	4699	831	PF01459	Porin – 3	276	29786	8.57	-0.192	Mitochondria
	15	Glyma.19G020100	*GmaVDAC19.1*	Chr19	reverse	2126365	2129292	2927	831	PF01459	Porin – 3	276	29705	9.06	-0.237	Mitochondria
G. soja	1	Glyso.01G155600	*GsoVDAC1.1*	Chr 1	forward	53847860	53851885	4025	831	PF01459	Porin – 3	276	29135	9.22	-0.103	Mitochondria
	2	Glyso.04G138200	*GsoVDAC4.1*	Chr 4	forward	43528144	43530466	2322	747	PF01459	Porin – 3	248	27064	7.73	-0.17	Mitochondria
	3	Glyso.05G026400	*GsoVDAC5.1*	Chr 5	forward	2584220	2586137	1917	738	PF01459	Porin – 3	245	26545	5.96	0.019	Mitochondria
	4	Glyso.06G174300	*GsoVDAC6.1*	Chr 6	reverse	17008501	17011143	2642	747	PF01459	Porin – 3	248	26783	8.81	-0.16	Mitochondria
	5	Glyso.08G261800	*GsoVDAC8.1*	Chr 8	reverse	41702181	41705132	2951	831	PF01459	Porin – 3	276	29804	7.07	-0.237	Mitochondria
	6	Glyso.09G194600	*GsoVDAC9.1*	Chr 9	forward	44020808	44025334	4526	831	PF01459	Porin – 3	276	29756	8.57	-0.188	Mitochondria
	7	Glyso.11G043700	*GsoVDAC11.1*	Chr 11	reverse	3634395	3638307	3912	831	PF01459	Porin – 3	276	29181	9.29	-0.08	Mitochondria

	8	Glyso.13G041200	*GsoVDAC13.1*	Chr 13	forward	15841924	15845030	3106	831	PF01459	Porin – 3	276	29777	9.05	-0.24	Mitochondria
	9	Glyso.13G065900	*GsoVDAC13.2*	Chr 13	forward	20474307	20479834	5527	834	PF01459	Porin – 3	277	29757	8.66	-0.159	Mitochondria
	10	Glyso.14G079400	*GsoVDAC14.1*	Chr 14	reverse	8484234	8487845	3611	834	PF01459	Porin – 3	277	29878	9.25	-0.191	Mitochondria
	11	Glyso.17G058900	*GsoVDAC17.1*	Chr 17	reverse	5012370	5018840	6470	834	PF01459	Porin – 3	277	29961	8.99	-0.178	Mitochondria
	12	Glyso.17G089500	*GsoVDAC17.2*	Chr 17	reverse	7806853	7808891	2038	738	PF01459	Porin – 3	245	26637	6.29	0.024	Mitochondria
	13	Glyso.18G100400	*GsoVDAC18.1*	Chr 18	forward	15915240	15917817	2577	831	PF01459	Porin – 3	276	29789	7.07	-0.208	Mitochondria
	14	Glyso.18G199200	*GsoVDAC18.2*	Chr 18	reverse	53550860	53555642	4782	831	PF01459	Porin – 3	276	29786	8.57	-0.192	Mitochondria
	15	Glyso.19G017500	*GsoVDAC19.1*	Chr 19	reverse	2100652	2103902	3250	831	PF01459	Porin – 3	276	29705	9.06	-0.237	Mitochondria
	1	Lj1g0025216	*LjaVDAC1.1*	Chr 1	forward	35009048	35014104	5056	831	PF01459	Porin – 3	276	29540	8.56	-0.131	Mitochondria
	2	Lj2g0020346	*LjaVDAC2.1*	Chr 2	reverse	2321010	2324669	3659	831	PF01459	Porin – 3	276	29139	9.13	-0.054	Mitochondria
	3	Lj2g0025788	*LjaVDAC2.2*	Chr 2	forward	16328032	16330866	2834	831	PF01459	Porin – 3	276	29714	8.57	-0.174	Mitochondria
L. japonicus	4	Lj4g0008003	*LjaVDAC4.1*	Chr 4	forward	23421330	23423360	2030	729	PF01459	Porin – 3	242	26730	7.7	-0.007	Mitochondria
	5	Lj4g0018809	*LjaVDAC4.2*	Chr 4	reverse	15869516	15878880	9364	1872	PF01459	Porin – 3	623	70492	8.96	-0.2	Mitochondria
	6	Lj4g0019132	*LjaVDAC4.3*	Chr 4	reverse	39564926	39567587	2661	831	PF01459	Porin – 3	276	29604	6.71	-0.151	Mitochondria
	7	Lj5g0023114	*LjaVDAC5.1*	Chr 5	forward	60557749	60559875	2126	843	PF01459	Porin – 3	280	29894	9.44	-0.148	Mitochondria
	1	Lup013425	*LanVDAC1.1*	Chr 1		22080858	22083836	2978	810	PF01459	Porin – 3	269	28830	7.05	-0.099	Mitochondria
	2	Lup001099	*LanVDAC3.1*	Chr 3		4866544	4870303	3759	834	PF01459	Porin – 3	277	29699	9.3	-0.212	Mitochondria
L. angustifolius	3	Lup004188.1	*LanVDAC5.1*	Chr 5		970961	973643	2682	831	PF01459	Porin – 3	276	29594	9.06	-0.211	Mitochondria
	4	Lup004097.1	*LanVDAC8.1*	Chr 8		10642106	10645067	2961	831	PF01459	Porin – 3	276	29703	8.57	-0.148	Mitochondria
	5	Lup005627.1	*LanVDAC17.1*	Chr 17		17996055	17997565	1510	732	PF01459	Porin – 3	243	26168	6.29	-0.035	Mitochondria
	6	Lup008309.1	*LanVDAC20.1*	Chr 20		18052403	18054425	2022	831	PF01459	Porin – 3	276	29615	9.06	-0.215	Mitochondria
	1	Medtr1g018205	*MtrVDAC1.1*	Chr 1	forward	5242716	5245592	2876	834	PF01459	Porin – 3	277	29708	9.03	-0.212	Mitochondria
	2	Medtr4g107840	*MtrVDAC4.1*	Chr 4	reverse	44694241	44696072	1831	735	PF01459	Porin – 3	244	26586	7.05	0.037	Mitochondria
	3	Medtr4g128300	*MtrVDAC4.2*	Chr 4	reverse	53417279	53422788	5509	840	PF01459	Porin – 3	279	30033	9.19	-0.262	Mitochondria
M. truncatula	4	Medtr5g015580	*MtrVDAC5.1*	Chr 5	reverse	5408078	5411450	3372	831	PF01459	Porin – 3	276	29478	9.4	-0.18	Mitochondria
	5	Medtr6g011390	*MtrVDAC6.1*	Chr 6	reverse	3207207	3211704	4497	837	PF01459	Porin – 3	278	29977	9.02	-0.182	Mitochondria
	6	Medtr7g009330	*MtrVDAC7.1*	Chr 7	reverse	2017987	2022950	4963	834	PF01459	Porin – 3	277	29667	8.91	-0.171	Mitochondria
	7	Medtr8g469150	*MtrVDAC8.1*	Chr 8	forward	25095913	25099359	3446	837	PF01459	Porin – 3	278	29847	7.06	-0.181	Mitochondria
P. vulgaris	1	Phvul.001G027200	*PvuVDAC1.1*	Chr 1	reverse	2417576	2420077	2501	834	PF01459	Porin – 3	277	29740	9.25	-0.222	Mitochondria
	2	Phvul.002G011300	*PvuVDAC2.1*	Chr 2	reverse	1219302	1222963	3661	831	PF01459	Porin – 3	276	29141	9.3	-0.068	Mitochondria

	3	Phvul.003G069300	*PvuVDAC3.1*	Chr 3	forward	10482086	10484512	2426	831	PF01459	Porin – 3	276	29631	7.1	-0.17	Mitochondria
	4	Phvul.003G146600	*PvuVDAC3.2*	Chr 3	reverse	35636493	35645175	8682	834	PF01459	Porin – 3	277	30004	8.86	-0.193	Mitochondria
	5	Phvul.003G192600	*PvuVDAC3.3*	Chr 3	forward	41657515	41659554	2039	738	PF01459	Porin – 3	245	26537	6.49	0.019	Mitochondria
	6	Phvul.008G043800	*PvuVDAC8.1*	Chr 8	forward	3732645	3736436	3791	831	PF01459	Porin – 3	276	29727	8.56	-0.159	Mitochondria
	7	Phvul.009G190000	*PvuVDAC9.1*	Chr 9	forward	28832072	28834273	2201	744	PF01459	Porin – 3	247	26994	8.51	-0.097	Mitochondria
P. sativum	1	Psat1g077520.1	*PsaVDAC1.1*	Chr 1		125452076	125456938	4862	1362	PF01459	Porin – 3	453	50136	9.4	-0.274	Mitochondria
	2	Psat2g171360.1	*PsaVDAC2.1*	Chr 2		409447609	409451531	3922	831	PF01459	Porin – 3	276	29571	9.33	-0.203	Mitochondria
	3	Psat3g195320.1	*PsaVDAC3.1*	Chr 3		417766932	417771773	4841	831	PF01459	Porin – 3	276	29597	9.11	-0.186	Mitochondria
	4	Psat4g053560	*PsaVDAC4.1*	Chr 4		87980607	87981933	1326	732	PF01459	Porin – 3	243	26595	8.67	0.002	Mitochondria
	5	Psat4g132360.1	*PsaVDAC4.2*	Chr 4		252322524	252325440	2916	831	PF01459	Porin – 3	276	29504	6.37	-0.097	Mitochondria
	6	Psat6g016200.1	*PsaVDAC6.1*	Chr 6		11720535	11722386	1851	834	PF01459	Porin – 3	277	29765	8.99	-0.183	Mitochondria
	7	Psat7g023480	*PsaVDAC7.1*	Chr 7		36236820	36238662	1842	948	PF01459	Porin – 3	315	34328	9.39	-0.041	Mitochondria
V. angularis	1	Vang01g18540	*VanVDAC1.1*	Chr 1		29044867	29047540	2673	834	PF01459	Porin – 3	277	29982	9.27	-0.206	Mitochondria
	2	Vang04g04860	*VanVDAC4.1*	Chr 4		6201042	6204927	3885	975	PF01459	Porin – 3	324	35653	9.66	-0.231	Mitochondria
	3	Vang07g02760	*VanVDAC7.1*	Chr 7		3012303	3022174	9871	843	PF01459	Porin – 3	280	29626	9.43	-0.079	Mitochondria
	4	Vang11g10150	*VanVDAC11.1*	Chr 11		14319483	14321335	1852	738	PF01459	Porin – 3	245	26641	6.12	-0.009	Mitochondria
	5	Vang11g14830	*VanVDAC11.2*	Chr 11		20037513	20047108	9595	894	PF01459	Porin – 3	297	32295	8.94	-0.24	Mitochondria
V. unguiculata	1	Vigun02g151400	*VunVDAC2.1*	Chr 2	forward	29837298	29841286	3988	831	PF01459	Porin – 3	276	29041	9.29	-0.042	Mitochondria
	2	Vigun03g212600	*VunVDAC3.1*	Chr 3	forward	35246669	35249039	2370	831	PF01459	Porin – 3	276	29532	7.1	-0.147	Mitochondria
	3	Vigun03g333100	*VunVERC3.2*	Chr 3	reverse	53109256	53112193	2937	738	PF01459	Porin – 3	245	26627	6.29	-0.013	Mitochondria
	4	Vigun03g382100	*VunVDAC3.3*	Chr 3	forward	58673993	58683800	9807	834	PF01459	Porin – 3	277	30069	8.87	-0.206	Mitochondria
	5	Vigun05g045100	*VunVDAC5.1*	Chr 5	forward	3727804	3731657	3853	831	PF01459	Porin – 3	276	29651	8.57	-0.172	Mitochondria
	6	Vigun08g030100	*VunVDAC8.1*	Chr 8	reverse	2746053	2748389	2336	834	PF01459	Porin – 3	277	29850	9.25	-0.193	Mitochondria
	7	Vigun09g078500	*VunVDAC9.1*	Chr 9	forward	9118152	9120175	2023	738	PF01459	Porin – 3	245	26921	8.4	-0.096	Mitochondria
V. radiata	1	Vradi04g08510	*VraVDAC4.1*	Chr 4		17018025	17021654	3629	900	PF01459	Porin – 3	299	32138	8.54	-0.13	Mitochondria
	2	Vradi06g14680	*VraVDAC6.1*	Chr 6		34522992	34525833	2841	747	PF01459	Porin – 3	248	26696	9.1	-0.216	Mitochondria
	3	Vradi07g05600	*VraVDAC7.1*	Chr 7		11915309	11917848	2539	831	PF01459	Porin – 3	276	29624	7.14	-0.159	Mitochondria
	4	Vradi07g15360	*VraVDAC7.2*	Chr 7		35643102	35644934	1832	738	PF01459	Porin – 3	245	26668	6.12	-0.014	Mitochondria
	5	Vradi07g22150	*VraVDAC7.3*	Chr 7		45043036	45051901	8865	1044	PF01459	Porin – 3	347	38205	7.11	-0.171	Mitochondria
	6	Vradi11g04110	*VraVDAC11.1*	Chr 11		3851631	3855392	3761	831	PF01459	Porin – 3	276	29117	9.3	-0.05	Mitochondria
